# Developing an expert consensus statement on emergency preparedness for cancer care delivery in Canada: a modified Delphi study

**DOI:** 10.1186/s13690-026-01845-y

**Published:** 2026-01-29

**Authors:** Hugh Andrew Jinwook Kim, Rui Fu, Pabiththa Kamalraj, Jonathan C. Irish, Frances C. Wright, Craig C. Earle, Apostolos Christopoulos, Kevin A. Hildebrand, Danny Enepekides, Julie Hallet, Antoine Eskander

**Affiliations:** 1 Department of Otolaryngology–Head and Neck Surgery, University of Toronto, 2075 Bayview Ave., M1-102, Toronto, ON M4N 3M5 Canada; 2 Departments of Community Health Sciences, Surgery & Oncology, Cumming School of Medicine, University of Calgary, Calgary, AB Canada; 3 Department of Surgery, University of Toronto, Toronto, ON Canada; 4 Department of Medicine, University of Toronto, Toronto, ON Canada; 5 Department of Surgery, Université de Montréal, Montréal, QC Canada; 6 Department of Surgery, Cumming School of Medicine, University of Calgary, Calgary, AB Canada

**Keywords:** Pandemic, COVID-19, Public health emergency, Consensus, Health policy, Cancer, Canada

## Abstract

**Background:**

In response to the COVID-19 pandemic, health organizations around the world including those in Canada have developed guidelines to allow for cancer care delivery amid a public health emergency. In Canada many of these guidelines were developed during the pandemic and at a time when there were limitations in knowledge on the impact of certain policies on patient care. Built upon this foundation we aimed to establish expert-informed, consensus-based policy recommendations to improve the preparedness and resilience of cancer systems for future public health emergencies, using lessons learned from the COVID-19 pandemic and focusing on the Canadian cancer care systems.

**Methods:**

We conducted a modified Delphi study using a two-round online survey administered to a Steering Committee composed of a purposefully sampled group of physicians and a patient advisor from across Canada. Participants rated their agreement with 23 Delphi statements across four domains: access to cancer surgery, virtual and home care, primary care, and institutional memory. These statements were developed through a focus group discussion with an Advisory Committee composed of Canadian health policy leaders during the COVID-19 pandemic and refined based on qualitative feedback. Consensus was defined a priori as greater than 70% agreement (6 or 7 on a 7-point Likert scale).

**Results:**

Seven of the 15 individuals invited to be panelists of the Advisory Committee and 16 of the 29 individuals invited to be panelists of the Steering Committee agreed to participate. Ten of the 23 statements reached consensus, including strengthening interhospital collaboration for surgical access, identifying health services appropriate for virtual care, expanding at-home cancer screening, and reducing administrative burden through artificial intelligence. In contrast, proposals related to primary care reform and infrastructure expansion did not achieve consensus. Qualitative responses revealed that disagreement mostly centered on perceived jurisdictional responsibility and resource constraints.

**Conclusion:**

We identified expert consensus on ten priorities that extend beyond immediate recovery and support system resilience. These recommendations may offer a foundation for healthcare leaders to impact national and international policymaking.

**Supplementary Information:**

The online version contains supplementary material available at 10.1186/s13690-026-01845-y.

**Table:** 

Text box 1. Contributions to the literature
• This modified Delphi study derives ten expert-consented policy priorities falling under four domains by which to strengthen pandemic preparedness for cancer systems in Canada. These results also have implications internationally. • Results of this study highlight the importance of developing and standardizing home/community-based care, including at-home cancer screening tests and nicotine replacement therapy, in the face of the next public emergency.
• At the institutional level, strengthening data infrastructure to protect against cyberattack and establishing agreements between hospitals to facilitate resource-sharing are highlighted. • To support vulnerable populations such as rural communities, expanding transportation, social and financial assistances may be crucial.

## Background

The coronavirus disease (COVID-19) pandemic has transformed cancer systems across jurisdictions, causing profound shifts in the processes and outcomes of cancer care at the population level [1–3]. In Ontario, the most populous province of Canada [4], a significant backlog of missed cancer diagnoses and elective cancer surgeries were observed during the early pandemic period [5, 6]. Reduced access to elective surgery has given rise to an increased use of neoadjuvant therapy including endocrine therapy and chemotherapy with unknown implications to treatment outcomes and patient well-being [7]. In-person oncologist visits were quickly replaced by telemedicine in some disease sites at the beginning of the pandemic, where audio-only phone visits were commonly used for a sustained period of time [8]. In the short term, these dramatic changes in the provision of cancer care do not appear to have affected patient survival [9], although modeling studies project the pandemic would cause excess cancer mortality in the next 5–20 years [10–12]. These abundant data are valuable only if they are being digested by decision makers and consensus can be reached on guidelines, to improve our system’s response during future public health emergencies.

In response to the COVID-19 pandemic, several organizations have developed guidelines for cancer care delivery. In Canada, many of these guidelines were developed amid the pandemic and at a time when there were limitations in knowledge on the impact of certain policies on patient care. For example, Ontario Health-Cancer Care Ontario, the provincial cancer authority, has published the *Pandemic Clinical Guideline for Patients with Cancer* [13], which outlines a framework for prioritizing cancer services during periods of system strain, and the *Person-Centred Virtual Cancer Care Clinical Guidance* [14], which provides dedicated recommendations on how to navigate virtual oncology encounters. At the national level, the Canadian Partnership Against Cancer (CPAC), an organization mandated by the Government of Canada to implement the Canadian Strategy for Cancer Control [15], has released the *Road to Recovery* framework [16], which focuses on restoring service disruptions and addressing backlogs in the immediate post-pandemic period. We aimed to build on this foundation to establish expert consensus on system-level priorities aimed at enhancing the resilience of cancer care delivery across Canada in future public health emergencies, using a replicable and scientifically robust methodology.

## Methods

### Study design

This study used a modified Delphi approach to reach expert consensus about policies to enhance pandemic preparedness in a cancer system with a focus on the Canadian cancer system. A Delphi method was chosen to enable a systemic process to gather expert opinion with a consensus-based approach [17]. The “modified” component refers to our engagement of an Advisory Committee to identify policy domains based on which we generated specific policy statements (i.e., Delphi consensus statements) prior to involving a Steering Committee to rank agreement with these statements [18]. This study was approved by the Research Ethics Board of Sunnybrook Health Sciences Centre (#5876). All panelists signed informed consent before participating. The survey rounds were conducted between August 2024 and February 2025, and results were reported following the recommendations for studies using a Delphi methodology [19] and the Consolidated criteria for reporting qualitative research guidelines [20].

### Expert panelists sampling and recruitment

For the Advisory Committee, we recruited via email invitations individuals from across Canada who were actual decision-makers and physician leaders during the COVID-19 pandemic. We intentionally invited these individuals as they had been intimately involved in policy planning during the pandemic and thereby were likely to continue having direct influence on developing policies for pandemic preparedness. Members of the Steering Committee included physicians in surgical oncology, medical oncology, radiation oncology, internal medicine, critical care medicine, urology, gastroenterology, general surgery and psychiatry, all members of the Advisory Committee, and a lived experience individual (henceforth, patient) advisor. Purposeful sampling was used to maximize diversity in gender, age, specialty, geographic location, and race/ethnicity when assembling both the Advisory and the Steering Committees. A patient advisor was recruited from Odette Cancer Centre’s Patient and Family Advisory Committee at Sunnybrook Health Sciences Centre.

### Identifying policy domains

An initial 90-minutes panel meeting was conducted with the Advisory Committee. A report of data regarding the pandemic impact on the Canadian cancer system, including an Executive Summary and diagrams, was generated based on prior population-level analyses, a rapid review of the literature, and discussion amongst the research team (Supplemental Appendix). This report was presented to the Advisory Committee prior to the meeting. Virtual and in-person formats were offered, and all panelists chose to attend virtually. One research team member (RF, Postdoctoral Fellow, Female) moderated the focus group discussion with the support of two other members HAJK, Surgery Resident, Male; PK, Research Assistant, Female) who took notes on non-verbal cues. During the discussion, panelists were asked to provide opinions regarding the report and input on (1) broad domains that warrant policy initiatives in the post-pandemic era (e.g., telemedicine, cancer screening, cancer surgery) and (2) specific policy items they would want us to address in the consensus statements. The meeting was audio recorded and then anonymized and transcribed verbatim to allow for a thematic analysis. Key themes were iteratively analyzed and identified by two team members (HAJK and RF), both have experience in qualitative data analysis, to form both broad policy domains and specific Delphi statements voiced by the Advisory Committee. Results of the thematic analysis were discussed amongst the research team and a list of draft statements were generated based on four broad policy domains: access to cancer surgeries, virtual and home care, primary care, and institutional memory. Draft statements were circulated to the Advisory Committee via email to collect their feedback; revisions were made to finalize the list of statements.

### Delphi survey

Two rounds of Delphi surveys were planned for the Steering Committee [21]. Each panelist received the panel material, including the Executive Summary with diagrams, electronically for review prior to survey administration. Panelists then received a link with instructions on how to complete the survey, administered using SurveyMonkey (www.surveymonkey.com). Data were collected and analyzed anonymously. During the first round, panelists were asked to rank agreement with each statement on a 7-point Likert scale. A space was assigned for comments on the existing statements and suggestion of additional statements. The first-round survey took place over three months, with two email reminders sent out in between. Rankings were summarized in three categories to facilitate analysis: strongly agree/agree (Likert scale 1–2), neutral (3–5), and strongly disagree/disagree (6–7). Statements for which ≥ 70% agreement was achieved were accepted for the final consensus and removed from the subsequent survey round. Statements for which ≥ 70% disagreement was achieved were dropped from the consensus. During the second round of survey, summarized group rankings from the previous round were provided and panelists were asked to rank each statement similarly to the first round using a 7-point Likert scale. Additional statements for which ≥ 70% agreement was achieved were accepted for the final consensus. The second-round survey took place over four weeks, with one email reminder sent out in between.

## Results

### Panelists

Of the 15 individuals representing decision-makers and clinical leaders during the COVID-19 pandemic invited to be panelists of the Advisory Committee, seven accepted the invitation to participate in the focus group discussion (Table 1). Of the 29 individuals invited to participate as panelists of the Steering Committee, sixteen signed the consent for participation and completed the first and second rounds of the Delphi survey (Table 2).

**Table 1:** Advisory committee panelists

Last name	First name	Leadership position	Specialty	Province of practice
Christopoulos	Apostolos	Dr Azar-Angélil Chair in Head and Neck Oncology, Université de Montréal	Surgical oncology	Quebec
Earle	Craig	Chief Executive Officer, Canadian Partnership against Cancer	Medical oncology	Ontario
Enepekides	Danny	Regional Surgical Oncology Lead, Ontario Health-Cancer Care Ontario	Surgical oncology	Ontario
Eskander	Antoine	Chief of Otolaryngology, Michael Garron Hospital	Surgical oncology	Ontario
Hildebrand	Kevin A	Head of Department of Surgery, Alberta Health Services Calgary Zone	Orthopedic surgery	Alberta
Irish	Jonathan C	Vice-President, Clinical, Cancer Programs, Ontario Health-Cancer Care Ontario	Surgical oncology	Ontario
Wright	Frances	Chief of Surgery, Sunnybrook Health Sciences Centre	Surgical oncology	Ontario

Of the 15 individuals we invited to be part of the Advisory Committee, seven (above) provided a written informed consent and participated in a 90-minutes focus group discussion held on Zoom on April 26, 2024

**Table 2:** Steering committee panelists

Last name	First name	Affiliated healthcare centre	Specialty	Province of practice
Bell	Gary	Patient advisor	-	Ontario
Brown	Carl J	St. Paul’s Hospital	Surgical oncology	British Columbia
Dahine	Joseph	Hôpital de la Cité-de-la-Santé (CISSS de Laval)	Critical care medicine	Quebec
Darling	Gail	QEII Health Sciences Centre	Thoracic surgery	Nova Scotia
Earle	Craig	Sunnybrook Health Sciences Centre	Medical oncology	Ontario
Feldman	Liane	Montreal General Hospital	General surgery	Quebec
Hallet	Juliet	Sunnybrook Health Sciences Centre	Surgical oncology	Ontario
Hanna	Timothy	Kingston Health Sciences Centre	Radiation oncology	Ontario
Hildebrand	Kevin A	Foothills Medical Center	Orthopedic surgery	Alberta
Irish	Jonathan C	University Health Network	Surgical oncology	Ontario
Ivers	Noah	Women’s College Hospital	Family medicine	Ontario
Krzyzanowska	Monika	Sunnybrook Health Sciences Centre	Medical oncology	Ontario
Singal	Rajiv	Michael Garron Hospital	Urology/Endourology	Ontario
Tinmouth	Jill	Sunnybrook Health Sciences Centre	Gastroenterology	Ontario
Urbach	David	Women’s College Hospital	Surgical oncology	Ontario
Wright	Frances	Sunnybrook Health Sciences Centre	Surgical oncology	Ontario

We invited 29 individuals to participate as a Steering Committee panelist for this Delphi study, including all of the Advisory Committee members, a patient advisor, and additional healthcare providers across Canada in a variety of clinical specialties. Sixteen individuals (above) signed a written consent form and provided responses to both Delphi survey rounds

*Abbreviations*: *QEII Health Sciences Centre* The Queen Elizabeth II Health Sciences Centre

### Delphi rounds

A total of 21 statements, covering four domains, were developed as the outcome of the Advisory Committee focus group discussion, and presented to the Steering Committee in the first round of survey (Table 3). There were five statements relating to access to cancer surgeries, six relating to virtual and home care, six relating to primary care, and four relating to institutional memory. Of these, ten (48%) reached consensus after the first round. No statements were dropped from the consensus due to disagreement. Thirteen statements, including the 11 not reaching consensus from the first-round and two additional statements panelists felt should be added, were presented to the Steering Committee in the second round of survey. No additional statements reached consensus after the second round.

**Table 3.**
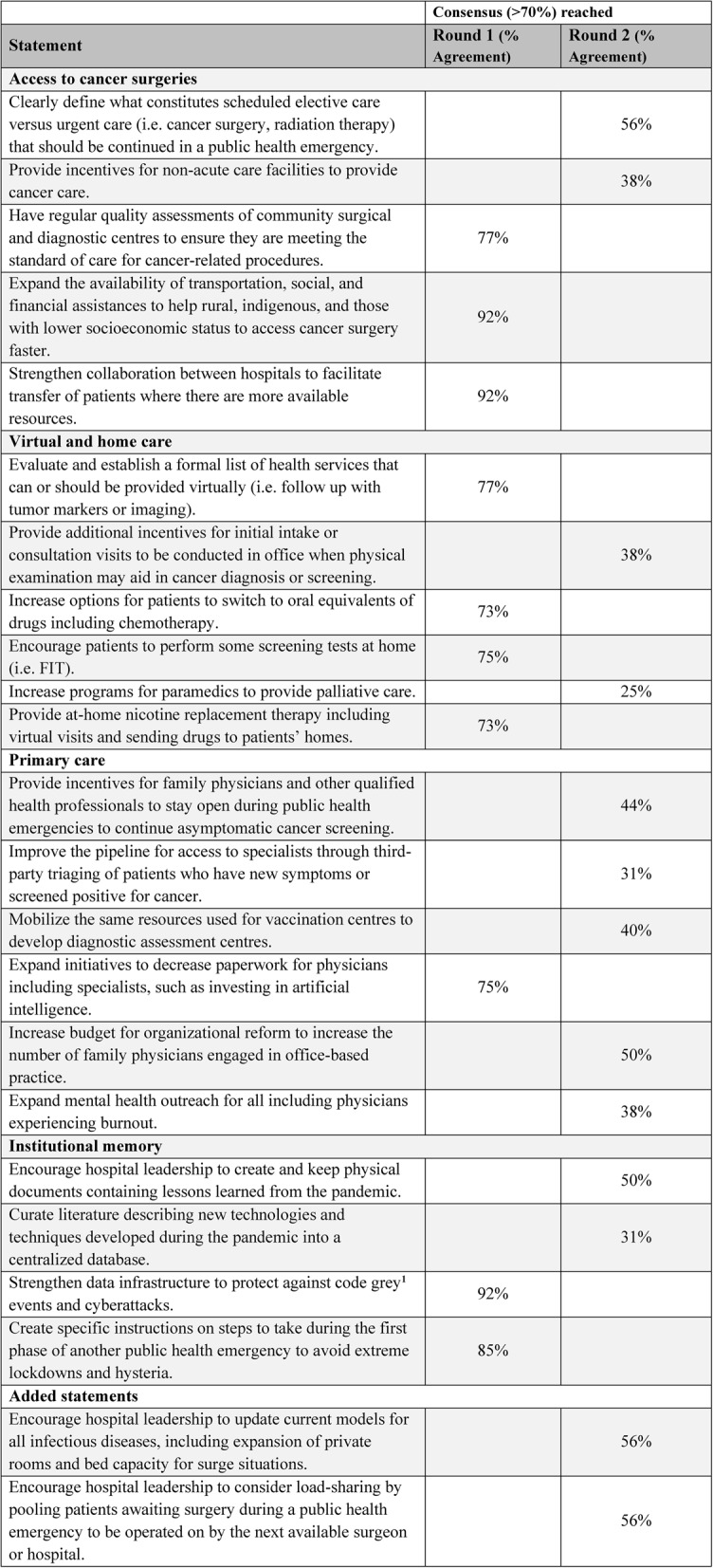
Results of deliberation

Participants ranked their agreements to the statements in Round 1 and 2. The statements were preceded by the phrase, “In the post-pandemic era, healthcare decision-makers and physicians should consider making the following changes to protect the delivery of cancer care:”

Code grey events refer to an urgent situation involving a loss of essential service or infrastructure failure in hospitals. These events include power outages, cyberattacks, medical gas supply issues, and other situations requiring immediate response to protect patient care

### Qualitative comments

Two panelists argued that the problem around family practice is not a matter of numbers and increasing the budget for more family physicians, but rather a matter of organizational reform to how primary care is delivered. Suggestions included increasing the proportion of family physicians engaged in office-based practice and proposing models of being assigned to a primary care practice based on neighbourhood or school district. One panelist strongly felt that telemedicine should be encouraged, which caused us to revise an initial statement discouraging the use of telemedicine for consultation visits, whereas another panelist commented that incentivizing in-person visits was unnecessary given that these should be standard whenever physical exams are necessary. One panelist commented that mental health counseling for physicians with burnout was generally unhelpful, compared to addressing the fundamental issues in their jobs that lead to burnout. Panelists commented that hospitals need to update current models for all infection control issues in and outside of a pandemic context, including private rooms for all patients and increased bed capacity for surge situations. They also recommended considering load-sharing by pooling patients awaiting surgery during a public health emergency to be operated on by the next available surgeon or hospital. Two new statements were added to the list based on this feedback but neither reached consensus. Other statements that reached consensus were accepted with minor revisions to terminology based on qualitative feedback, which we felt would not influence other panelists’ agreement on the statement.

### Final list of consensus statements

1. Evaluate and establish a formal list of health services that can or should be provided virtually (i.e. follow up with tumor markers or imaging).
2. Increase options for patients to switch to oral or subcutaneous equivalents of drugs including chemotherapy that can be delivered at home.
3. Provide encouragement, develop technologies, and remove barriers for patients to perform screening tests at home.
4. Provide at-home nicotine replacement therapy including virtual visits and sending drugs to patients’ homes.
5. Expand initiatives to decrease paperwork for physicians including specialists, such as investing in artificial intelligence.
6. Have regular quality assessments of community surgical and diagnostic centres to ensure they are meeting the standard of care for cancer-related procedures.
7. Expand the availability of transportation, social, and financial assistances to help rural, indigenous, and those with lower socioeconomic status to access cancer therapies (i.e. surgery or radiation) faster.
8. Have emergency preparedness agreements between hospitals to facilitate transfer of patients where there are more available resources.
9. Strengthen data infrastructure to protect against code grey events (an urgent situation involving a loss of essential service or infrastructure failure in hospitals) including power outages, cyberattacks, medical gas supply issues, and other emergencies.
10. Create specific instructions on steps to take during the first phase of another public health emergency to avoid extreme lockdowns and hysteria.

## Discussion

This modified Delphi study achieved expert consensus on 10 of 23 statements regarding proposed changes to the delivery of cancer care to enhance pandemic preparedness. Four were in the domain of virtual and home care, one in the domain of primary care, three in the domain of access to cancer surgeries, and two in the domain of institutional memory. All ten statements reached consensus during the first round of the Delphi survey. No further statements reached consensus in the second round despite incorporating panelists’ suggestions.

That four statements which reached final consensus were in the domain of virtual and home care highlights the widespread adoption and acceptance of virtual healthcare delivery, a practice initially born out of necessity during a period of lockdowns and social distancing [8, 22, 23]. While most agreed with the usefulness of defining a formal list of health services that can or should be provided virtually, opinions were divided on the idea of providing additional incentives for initial consultation visits to be conducted in-office where physical examination may be beneficial and, in many cases, necessary. Certainly, there are select cancer types where diagnosis and follow up are made primarily through imaging and tumor markers, such as hepatocellular carcinoma, germ cell tumors, and ovarian cancer, assuming image-guided biopsies can also be coordinated prior to the initial consultation [24]. Physicians should continue to exercise judgment on when an in-person encounter may enhance the therapeutic relationship, influence patient-centred decision-making, or potentially miss important clinical findings that may affect treatment decisions.

Access to cancer surgeries emerged as another key domain, with three statements reaching consensus. These had an emphasis on reducing structural barriers to improve equitable access to surgical care, from both the standpoint of bridging economic and social barriers, and equitable sharing of regional hospital resources. Agreement with these statements is not surprising as it has been widely understood that the pandemic has had disproportionately impacted people based on socioeconomic, racial/ethnical, and geopolitical factors [23, 25, 26].

The one statement reaching consensus in the domain of primary care related to the use of artificial intelligence to improve productivity by decreasing paperwork. While this issue is particularly relevant to primary care, it also affects specialists including oncologists, where high patient volumes and complex investigations create significant administrative burden. Thus, more panelists may have been able to relate personally to the statement. Ambient artificial intelligence scribes have seen growing adoption [27]. Feedback from physicians and patients have included the increased ability to have personal, meaningful, and effective interactions during the therapeutic encounter [28]. Notably, a new leadership position has recently been established at Ontario Health specifically focused on addressing this issue, further highlighting its importance as an area for system improvement. By contrast, the other statements failed to reach consensus despite undergoing revisions. This highlights an important aspect of our study that, while primary care is critical to cancer screening and referrals, its unique challenges may not have been fully captured in a panel primarily composed of hospital-based specialists.

There were two statements reaching consensus in the domain of institutional memory. These both had a greater focus on strengthening infrastructure and protocols to prevent and mitigate the impact of future unexpected emergencies. This may reflect a visceral response in our respondents as the COVID-19 pandemic served as a wakeup call to the global community highlighting how unprepared health systems were to manage such crises [29].

The CPAC has previously published a national framework in response to the widespread disruptions to cancer care caused by the COVID-19 pandemic [16]. As echoed in our study, the CPAC’s *Road to Recovery* report emphasizes the disruptions in cancer care secondary to postponed diagnostic exams and treatments leading to a backlog of undiagnosed and untreated cases. Their document focused on strategies for pandemic response and systems recovery in the immediate post-pandemic period. Our study adds upon the CPAC’s previous work by developing expert consensus on how to build resilience into the systems that deliver cancer care in Canada and increase preparedness for the next public health crisis. While Ontario Health’s virtual care guideline focused on principles and logistical guidance for delivering virtual oncology care [14], our Delphi process achieved consensus on the need to devise a formal list of cancer services that can and should be provided virtually.

Strengths of our study include its structured modified Delphi methodology, which is rigorous and valuable in areas with limited empirical data [21]. The purposefully sampled Advisory and Steering Committees included a heterogeneous, multidisciplinary selection of health policy decision-makers/advisors, physicians and a patient advisor. The relatively low recruitment rate of the Advisory Committee (7/15 or 47%) was a limitation of this study as representation of non-Ontario panelists was low (2/15 or 13%) although Ontario, the most populous province of Canada, did account for more than 40% of the Canadian population [4]. We achieved a slightly higher recruitment rate for the Steering Committee (16/29 recruited or 55%) and also a better capturing of panelists from outside of Ontario (British Columbia, Quebec, Nova Scotia, and Alberta). The physician panelists had a wide range of specialities including oncology, surgery, critical care medicine, urology, gastroenterology, and family medicine which were key to this Delphi study. However, we failed to recruit any psychiatrists or psychologists despite inviting two such individuals to participate. We also did not recruit any non-physician healthcare professionals such as nurses, physician assistants, and social workers despite their important roles in shaping and executing new processes of care during the pandemic. We made this decision intentionally to only recruit a relatively small cohort of individuals who had already been heavily involved in the actual policy planning and decision-making during the pandemic and would likely to have continuing impact on policies post-pandemic. Future expert consensus efforts should intentionally recruit non-physician healthcare providers and hospital administrators to incorporate their suggestions and viewpoints to policy formulation.

Whether viewed as a limitation or a reflection of strong initial alignment, there were few additional qualitative comments that emerged from the first round of the Delphi survey to improve or expand the existing list of statements, and overall consensus was more divided in the second round of survey compared to the first round. This may reflect survey fatigue leading to panelists to respond more neutrally [30]. We also decided not to advance to a real-time roundtable discussion to explore disagreements, given the logistical difficulty in finding a mutually convenient time for all panelists, and risk of introducing bias if only available panelists were invited.

While the consensus statements developed through our Delphi process represent expert-informed system-level priorities for cancer care preparedness, their implementation will require coordinated planning across provincial jurisdictions, institutional leaders, and clinical teams. Research in implementation science has shown that uptake of guidelines and consensus recommendations is often challenged by contextual factors such as limited resources, lack of awareness or resistance to change from stakeholders, and the complexity of healthcare delivery environments [31–33]. To address these challenges, implementation of our consensus statements should follow a structured, barrier-informed approach that incorporates stakeholder co-design, resource planning, and implementation strategies tailored to the local context of each care setting. Future research could explore the feasibility and real-world impacts of operationalizing these statements through pilot programs or integration into existing pandemic preparedness and recovery frameworks.

## Conclusions

This modified Delphi study has produced expert consensus on key policy priorities to strengthen Canadian systems for cancer care delivery in the post-pandemic era. Consensus was reached on ten statements distributed across domains of virtual and home care, access to cancer surgeries, primary care, and institutional memory. These expert-informed recommendations may offer a foundation for healthcare leaders to engage stakeholders and take action to make long-lasting changes to cancer care and emergency preparedness across Canada and also have implications internationally.

## Supplementary Information

Supplementary Material 1.

## Data Availability

Panel material, including the Executive Summary and diagrams, is included in the Supplementary Appendix. The audio recording analysed during the current study is not publicly available because they could include identifiers.

## Abbreviations

COVID-19: Coronavirus disease
CPAC: Canadian Partnership Against Cancer
QEII Health Sciences Centre: The Queen Elizabeth II Health Sciences Centre
