# Supplementary material for: Developing an expert consensus statement on emergency preparedness for cancer care delivery in Canada: a modified Delphi study
Source: Arch Public Health. 2026 Jan 29;84:44. doi: 10.1186/s13690-026-01845-y (PMC12924313; doi:10.1186/s13690-026-01845-y)
Supplement: Supplementary file 1 — Supplementary Material 1. [file 13690_2026_1845_MOESM1_ESM.pdf]

COVID-19 IMPACT

Ontario's cancer services delivery and outcomes

During the first week of COVID-19

- 57% ↓ elective cancer surgical volume
- 12% ↓ cancer diagnostic imaging
- 47% ↓ in-person visits accompanied by a 5515% ↑ virtual visits at cancer diagnosis
- 93% ↓ bilateral mammography for breast screening
- 35% ↓ cancer incidence detection volume

Backlogs

- Missed cancer surgery cases = 66,376 (as of Aug 2021)
- Cancer incidence cases = 16,192 (as of Oct 2021)

For patients newly diagnosed with cancer during COVID-19

- ↑ in use of nonsurgical therapy as first treatment
- 12%-16% ↓ in wait times for patients able to get treated within 6 months of diagnosis
- ↑ in use of neoadjuvant chemotherapy
- 1-year overall survival comparable to those diagnosed with cancer in past 2 years, except Melanoma

Inequalities noted before and after COVID with no significant shifts

- Average-risk women who were younger, immigrants, and living in more materially deprived areas had lower use of bilateral mammography for breast screening
- New cancer patients who were immigrants or living in more materially deprived areas less likely to receive surgery as first cancer treatment
- Material deprivation also associated with a lower receipt of surgery following chemotherapy

Executive Summary

The COVID-19 pandemic has resulted in significant shifts in the processes and outcomes of cancer care in Ontario. Since March 2021, we have conducted a suite of population-based cohort studies using ICES data. Our findings suggest that during the first week of COVID-19 (the week of March 15, 2020), Ontario has experienced a:

- **57% ↓** elective cancer surgical volume.[1,2]
- **12% ↓** cancer diagnostic imaging (ultrasound ↓ 32%, CT ↓10%, MRI: no change).[3]
- **47% ↓** in-person visits accompanied by a 5515% ↑ virtual visits at cancer diagnosis.[3] In the general population, these changes were 65% ↓ for in-person visits and 2130% ↑ for virtual visits.[4]
- **93% ↓** bilateral mammography for breast screening.[5]
- **35% ↓** cancer incidence detection volume.[6,7]

As of August 2021, we estimated Ontario has cumulated a backlog of **66,376 missed cancer surgery cases**, and as of Oct 2021, the **backlog of cancer incidence cases amounts to 16,192**.[2,7]

For Ontario patients newly diagnosed with cancer during the pandemic, our findings suggest:

- an overall **shift towards using nonsurgical therapy** as the first treatment (30% ↑ chemotherapy-first, 13% ↑ radiation-first, 9% ↓ surgery-first).[8]
- **12-16% ↓ wait times** for those able to get treated within 6 month of diagnosis.[9]
- ↑ neoadjuvant chemotherapy.[10]
- **1-year overall survival was comparable** to those diagnosed with cancer in past 2 years, except for Melanoma that showed worse survival during the pandemic.[11]

Inequalities were noted both before and after the start of COVID in Ontario without any significant shift in magnitude:

- Average-risk women who were younger, immigrants, and living in more materially deprived areas had a lower use of bilateral mammography for breast screening.[5]
- New cancer patients who were immigrants or living in more materially deprived areas were less likely to receive surgery as their first cancer treatment.[9] Material deprivation was also associated with a lower receipt of surgery following chemotherapy.[10]

Similar patterns of COVID-19 impact on cancer care were observed in other countries. In terms of cancer screening and diagnosis, studies have reported:

- Between January and October 2020, overall 46.7% ↓ breast cancer screening, 44.9% ↓ colorectal cancer screening, 51.8% ↓ cervical cancer screening, compared with the previous period.[12]
- In 2020, 73.4% ↓ cervical cancer diagnoses in Portugal, 62% ↓ colorectal cancer diagnoses in Italy.[13]
- Between 2019-2021, 18-29% ↓ breast cancer diagnoses.[13]
- Estimated up to 63,229 years of life lost due to delays in cancer diagnosis during the first COVID-19 lockdown in the UK.[13]

- Estimated deaths up to 65,756, 32,644, 95,195, and 155,238 from breast, esophageal, lung, and colorectal cancer globally secondary to delayed cancer screening.[13]
- In Australia, 8% ↓ cancer-related diagnostic procedures.[14]
- In the US between March 1 to April 18, 2020, 46% ↓ new diagnoses of breast, colorectal, lung, pancreatic, gastric, and esophageal cancers.[14]
- In the Netherlands during February to August 2020, 67% ↓ screen-detected breast cancers, resulting in an estimated 2000 delayed detections.[14]
- Diagnostic delays in England are estimated to lead to up to 3621 additional deaths within 5 years across breast, colorectal, lung, esophageal cancers.[14]
- 26% ↓ outpatient visits.[15]

In terms of cancer treatment, studies have reported disturbances on both patient and institutional levels:

- 77.5% of patients surveyed reported interruption in any stage of cancer treatment. The observed number was 26.3% in three longitudinal studies.[16]
- Between January and October 2020, 33.9% ↓ overall cancer surgical treatments and 12.6 ↓ medical treatments, compared with the previous period.[13]
- 38% cancelled cancer surgeries worldwide.[17]
- In Australia, 9% ↓ therapeutic cancer procedures.[14]
- Delays in surgery (50%), systemic therapy (53.8%), radiotherapy (56.7%).[15]
- 57.1% ↓ surgical capacity, favouring systemic therapy or hypofractionated radiotherapy.[15]
- 79% of centres surveyed reported disruption in the supply chain, 36% reported medicine shortage leading to modification of chemotherapy regimens, and 43% reported reduced access to anticancer medication.[16]
- 60% of centres surveyed reported reduced personnel, in some cases by up to 50%.
- 30% ↓ cancer-related hospitalizations.[16]
- 65% ↓ clinical trial activities.[16]
- 65% of cancer patients had changes in treatment plans, such as non-surgical treatment over surgical treatments.[13]
- 88% of centres globally reported reduction in their usual level of cancer care.[14]

Mammography for average-risk women

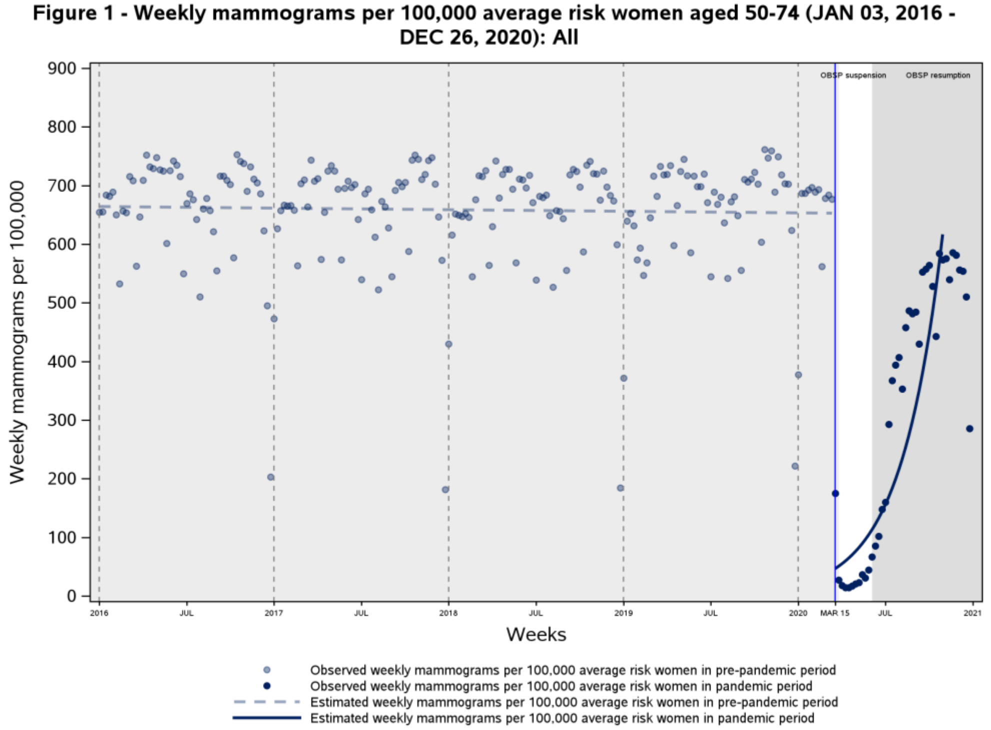

Cancer diagnostic imaging

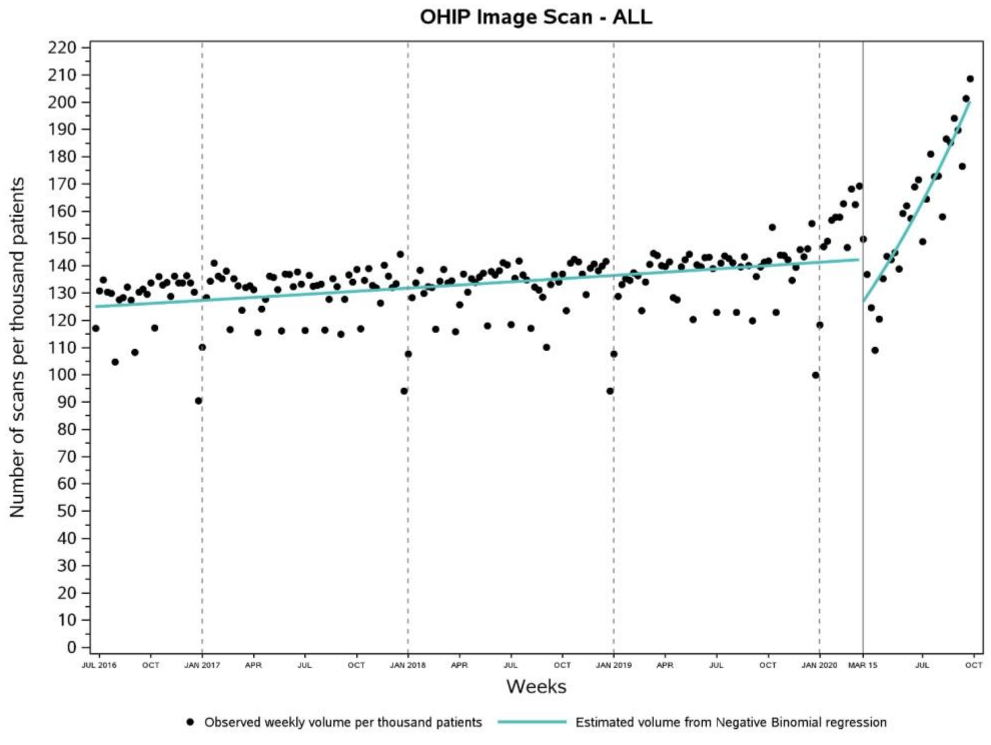

Physician visits – general population

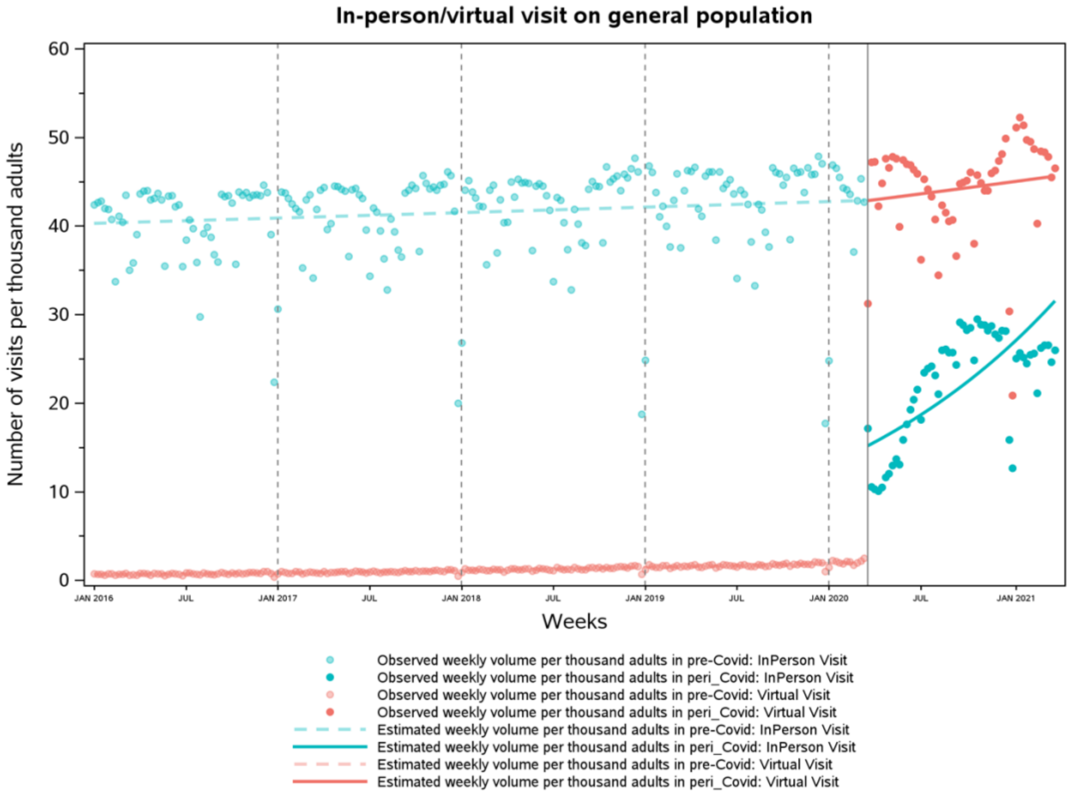

Physician visits – new cancer patients

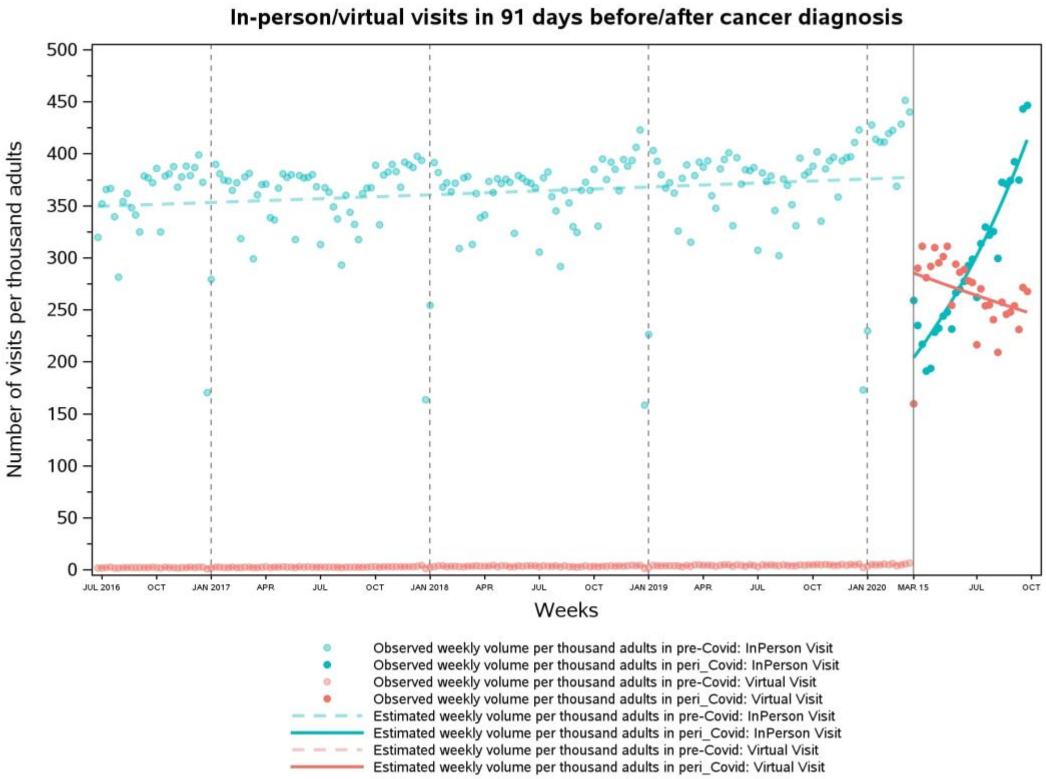

Cancer incidence

Figure 1 - Weekly Cancer Incidence (SEP 25 2016 - OCT 02 2021)

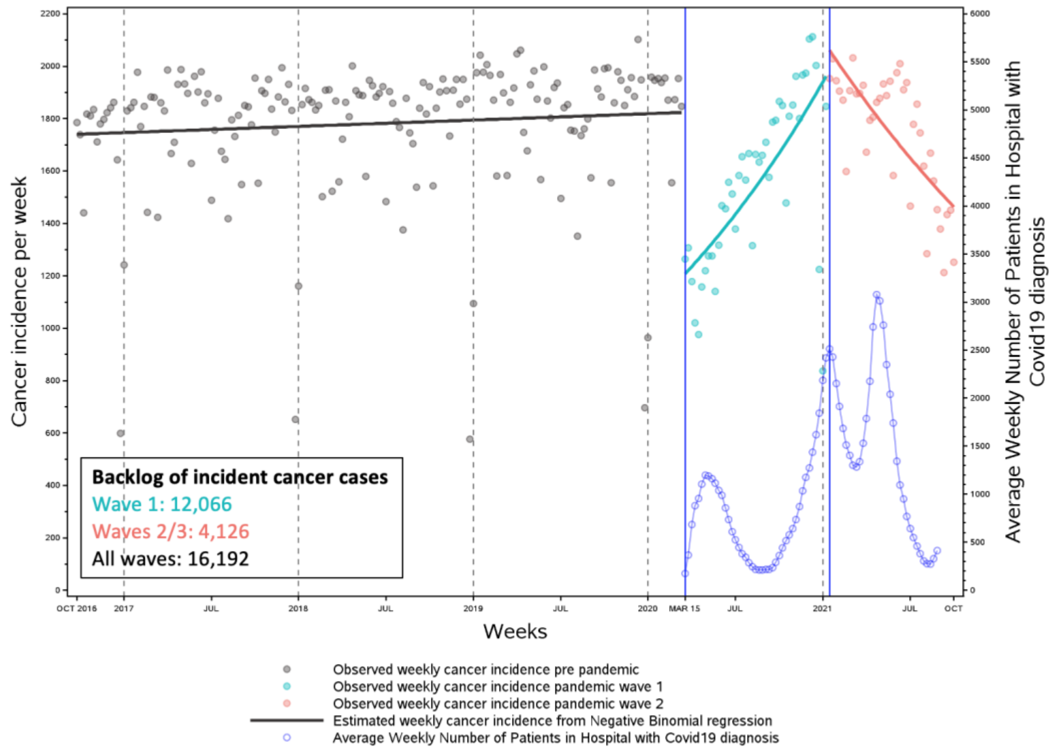

Cancer elective surgery

Figure 1 - Weekly Cancer-Directed Surgeries January 2018 - August 2021

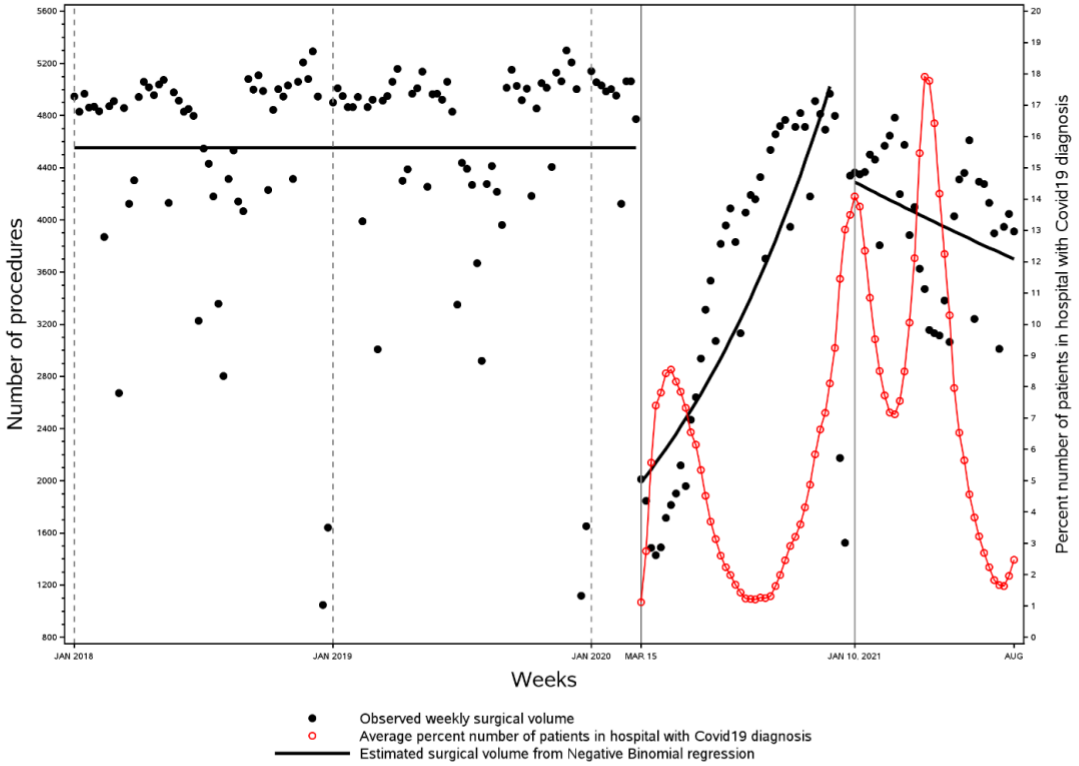

Cancer treatment modality: population-level trend

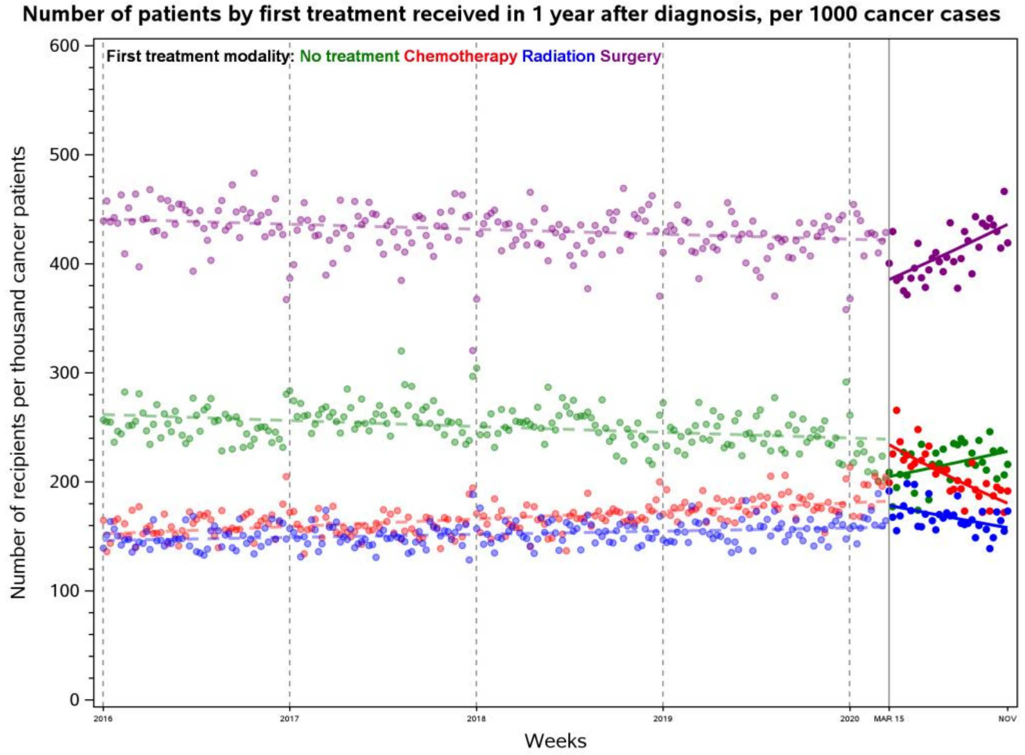

Cancer treatment modality: Individual-level probability

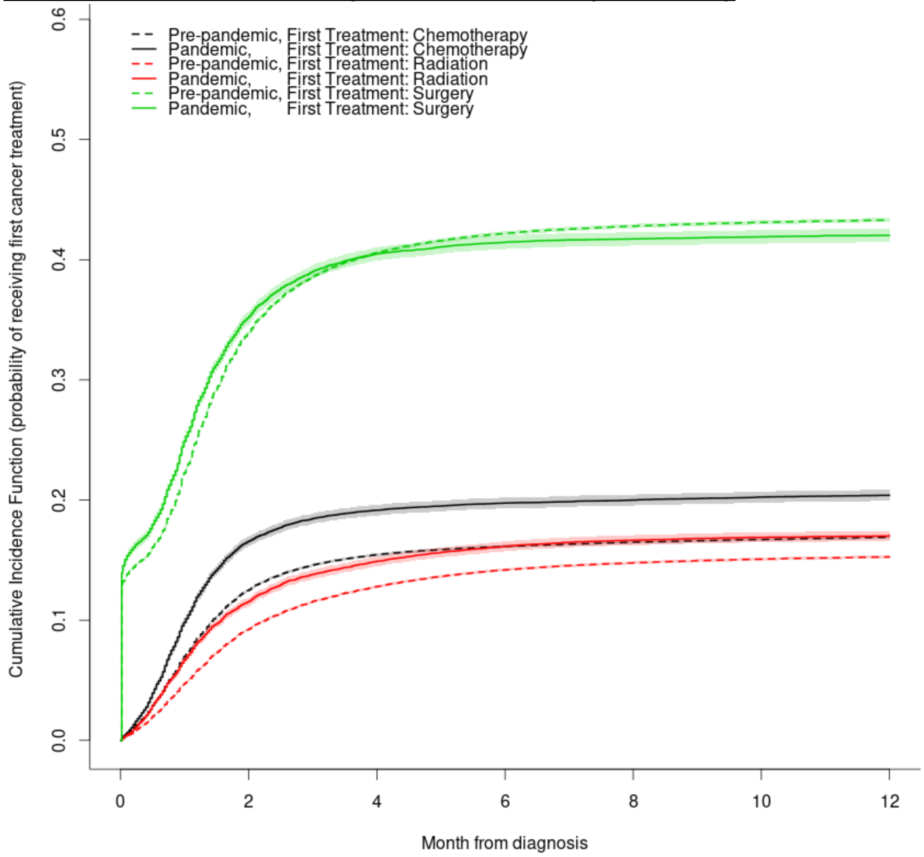

Cancer 1-year survival

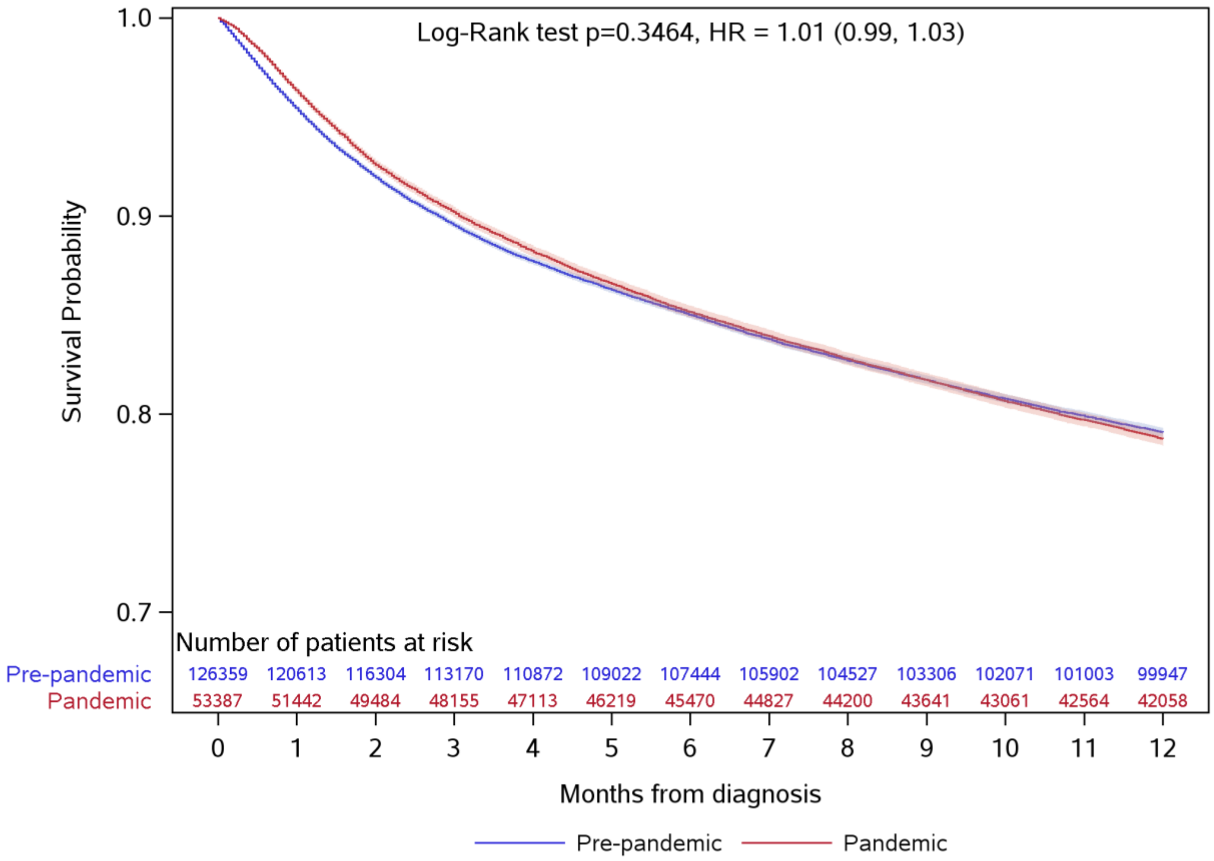

References:

1. Eskander A, Li Q, Hallet J, Coburn N, Hanna TP, Irish J, et al. Access to Cancer Surgery in a Universal Health Care System During the COVID-19 Pandemic. JAMA Netw Open. 2021;4(3):e211104.
2. Fu R, Kamalraj P, Li Q, Hallet J, Gomez D, Sutradhar R, et al. The Changing Face of Cancer Surgery During Multiple Waves of COVID-19. JNCI Cancer Spectr. 2022;6(5).
3. Fu R, Sutradhar R, Li Q, Hanna TP, Chan KKW, Coburn N, et al. Imaging and physician visits at cancer diagnosis: COVID -19 pandemic impact on cancer care. Cancer Med. 2022.
4. Fu R, Sutradhar R, Li Q, Eskander A. Virtual and in-person visits by Ontario physicians in the COVID-19 era. J Telemed Telecare. 2022:1357633X221086447.
5. Fu RD, Anna; Sutradhar, Rinku; Li, Qing; Hallet, Julie; Coburn, Natalie; Lapointe-Shaw, Lauren; Hong, Nichole J. Look; Karam, Irene; Krzyzanowska, Monika; Tinmouth, Jill; Eskander, Antoine; Rabeneck, Linda. COVID-19 pandemic impact on screening mammography participation: a population-based study. Preventative Medicine. 2023; Under submission since April 2023.
6. Eskander A, Li Q, Yu J, Hallet J, Coburn NG, Dare A, et al. Incident Cancer Detection During the COVID-19 Pandemic. J Natl Compr Canc Netw. 2022;20(3):276-84.
7. Fu R, Sutradhar R, Li Q, Hanna TP, Chan KKW, Irish JC, et al. Incident Cancer Detection During Multiple Waves of COVID-19: The Tsunami After the Earthquake. J Natl Compr Canc Netw. 2022;20(11):1190-2.
8. Fu R, Sutradhar R, Li Q, Hanna TP, Chan KKW, Irish JC, et al. Association between the COVID-19 pandemic and first cancer treatment modality: a population-based cohort study. CMAJ Open. 2023;11(3):E426-e33.
9. Fu R, Sutradhar R, Li Q, Hanna TP, Chan KKW, Irish JC, et al. Timeliness and Modality of Treatment for New Cancer Diagnoses During the COVID-19 Pandemic in Canada. JAMA Netw Open. 2023;6(1):e2250394.
10. Fu R, Sutradhar R, Dare A, Li Q, Hanna TP, Chan KKW, et al. Cancer Patients First Treated with Chemotherapy: Are They More Likely to Receive Surgery in the Pandemic? Curr Oncol. 2022;29(10):7732-44.
11. Fu R, Sutradhar R, Li Q, Kamalraj P, Dare A, Hanna TP, et al. Early survival for patients newly diagnosed with cancer during COVID-19 in Ontario, Canada: A population-based cohort study. Cancer Med. 2023;12(10):11849-59.
12. Teglia F, Angelini M, Astolfi L, Casolari G, Boffetta P. Global Association of COVID-19 Pandemic Measures With Cancer Screening: A Systematic Review and Meta-analysis. JAMA Oncology. 2022;8(9):1287-93.
13. Muka T, Li JJX, Farahani SJ, Ioannidis JPA. An umbrella review of systematic reviews on the impact of the COVID-19 pandemic on cancer prevention and management, and patient needs. eLife. 2023;12:e85679.
14. Milch V, Nelson AE, Austen M, Hector D, Turnbull S, Sathiaraj R, et al. Conceptual Framework for Cancer Care During a Pandemic Incorporating Evidence From the COVID-19 Pandemic. JCO Global Oncology. 2022(8):e2200043.
15. Powis M, Milley-Daigle C, Hack S, Alibhai S, Singh S, Krzyzanowska MK. Impact of the early phase of the COVID pandemic on cancer treatment delivery and the quality of cancer care: a scoping review and conceptual model. Int J Qual Health Care. 2021;33(2):mzab088.

16. Riera R, Bagattini Â M, Pacheco RL, Pachito DV, Roitberg F, Ilbawi A. Delays and Disruptions in Cancer Health Care Due to COVID-19 Pandemic: Systematic Review. JCO Glob Oncol. 2021;7:311-23.
17. Mohseni Afshar Z, Hosseinzadeh R, Barary M, Ebrahimpour S, Alijanpour A, Sayad B, et al. Challenges posed by COVID-19 in cancer patients: A narrative review. Cancer Medicine. 2022;11(4):1119-35.
